# Occupational Stress and the Quality of Life of Nurses in Infectious Disease Departments in China: The Mediating Role of Psychological Resilience

**DOI:** 10.3389/fpsyg.2022.817639

**Published:** 2022-03-21

**Authors:** Jiaran Yan, Chao Wu, Yanling Du, Shizhe He, Lei Shang, Hongjuan Lang

**Affiliations:** ^1^Nursing Department, Fourth Military Medical University, Xi’an, China; ^2^Department of Health Statistics, Fourth Military Medical University, Xi’an, China

**Keywords:** occupational stress, quality of life, psychological resilience, nurse, infectious disease departments

## Abstract

**Aim:**

We aim to explore the impact of occupational stress on the quality of life of nurses in infectious disease departments and to explore the mediating role of psychological resilience on this impact.

**Background:**

Sudden public health events and the prevalence of infectious diseases give nurses in infectious disease departments a heavy task load and high occupational stress, which can affect their quality of life, and which is closely related to the quality of clinical care they provide. There are few existing studies on occupational stress, psychological resilience, and the quality of life of nurses in infectious disease departments.

**Methods:**

We collected data from infectious-disease-specialized hospitals or infectious disease departments of general hospitals in China. In total 1,536 nurses completed questionnaires: the Effort-Reward Imbalance Questionnaire, the Connor-Davidson Resilience Scale, and the World Health Organization Quality of Life Brief Scale. We use a structural equation model to test the mediating role of the psychological resilience in the relationship between occupational stress and quality of life.

**Results:**

Among 1,536 participants, 88.2% experienced an effort-reward imbalance. The average scores for psychological resilience and quality of life were 56.06 (*SD* = 14.19) and 51.80 (*SD* = 8.23), respectively. Our results show that occupational stress is negatively correlated with psychological resilience (*r* = −0.28, *p* < 0.01) and quality of life (*r* = −0.44, *p* < 0.01). In addition, we find that psychological resilience is positively correlated with quality of life (*r* = 0.55, *p* < 0.01) and that the indirect effect of occupational stress on quality of life through psychological resilience is significant (β = −0.036, 95% *CI*: 0.027 to 0.426), indicating at least a partial mediating role of psychological resilience.

**Conclusion:**

A high proportion of nurses in infectious disease departments felt that their jobs’ effort-reward imbalance was high. These nurses’ scores for psychological resilience were in the middle level among Chinese people generally, but their quality of life was lower than the Chinese norm. We conclude that occupational stress has an important impact on their quality of life, and psychological resilience plays a partial mediating role on this impact.

**Implications for Nursing Management:**

Hospital managers can benefit from paying attention to the occupational stress of nurses and helping to improve the quality of life of nurses by alleviating this occupational stress and improving psychological resilience.

## Introduction

In recent years, the incidence of HIV/AIDS, syphilis, viral hepatitis, and other infectious diseases have all been on the rise, and there have been occasional outbreaks of unknown viruses such as SARS, influenza and COVID-19 that have seriously endangered human life and health (Smith et al., 2014; Vinkers et al., 2020; Wiersinga et al., 2020). This has had a major impact on health care, and when the epidemic broke out, nurses in infectious disease departments played an important role in the front line of medical aid. Infectious diseases departments are places where patients with infectious diseases are treated intensively, and many individuals, including those who work in these departments are afraid of infectious diseases that are highly contagious, have the potential to become epidemics, and are potentially fatal (Jung et al., 2015).

In addition, the high risk of occupational exposure to nurses and their anxiety about potential medical accidents combined with heavy work tasks can bring great psychological pressure to the nurses in infectious disease departments and affect their quality of life (Ding et al., 2018). The World Health Organization defines quality of life as the experience of individuals in different cultures and value systems of their living conditions in relation to their purposes, expectations, standards, and concerns (WHO, 1993; Lucas-Carrasco, 2012). At present, scholars have carried out some studies on the influencing factors of nurses’ quality of life (Kumar et al., 2018; Huang et al., 2020) and have found that occupation-related factors can be important (Ruiz-Fernández et al., 2020a).

Nurses generally have a high degree of occupational stress (Molina-Praena et al., 2018; Faraji et al., 2019). Occupational stress refers to physical and psychological stress caused by work or work-related factors, the physical or psychological stress response caused when the demands of a job exceed the ability of an employee (Pyshnov, 2003), and we can use the effort-reward imbalance scale to evaluate occupational stress (Siegrist and Dragano, 2008; Jachens and Houdmont, 2019). This is important because under long-term stress, physical health is prone to decline, leading to an increase in the prevalence of hypertension, anxiety, depression, and other diseases (Buselli et al., 2020). This can cause nurses to lack the energy to deal with the challenges of work and life affairs effectively, which in turn can lead to the reduction of work efficiency and happiness in their lives.

Psychological resilience is a person’s ability to cope with stress in a healthy way and achieve goals with minimal mental and physical cost (Sisto et al., 2019). As a positive psychological resource for individuals to cope with stress, psychological resilience can help individuals effectively reduce the negative impact of stress, and it can have positive impacts on individual social adaptability and physical and mental health (Cooper et al., 2020) as well. Previous studies have found that occupational stress is negatively correlated with psychological resilience (Wang et al., 2017). When individuals are under high occupational stress, their mental resilience is generally poor, and their ability to alleviate the negative effects of stress is also generally poor. When psychological resilience is high, however, people tend to have more energy and enthusiasm with which to regulate their emotions. They also tend to use a variety of positive resources to relieve work pressure and life troubles, which can have a positive role in promoting work quality and overall happiness.

Therefore, we speculate that there is a correlation between occupational stress, psychological resilience, and quality of life and that psychological resilience may indeed be a mediating mechanism between occupational stress and quality of life. However, there are few relevant studies on nurses in infectious diseases departments specifically so the purposes of this study are twofold. First, we explore the relationship between the level of occupational stress and quality of life of nurses in infectious disease departments. Second, we explore whether there is a mediating effect of psychological resilience on this relationship.

## Literature Review and Hypotheses

### Occupational Stress and Quality of Life

Social support, stress response, and other stress-related factors have recently become the focus of research on the influencing factors of nurses’ quality of life (Kowitlawkul et al., 2019; Vafaei et al., 2020), and occupational stress in particular is considered to be an important factor that affects quality of life (Chegini et al., 2019). Long-term, high-intensity occupational stress can produce a series of stress reactions that affect the physical and mental health of many types of professionals (Nochaiwong et al., 2020). Because of the high risk of occupational exposure to nurses in infectious disease departments, nurses often have to deal with long-term, high-intensity work in addition to frequent staff shortages (Drennan and Ross, 2019). Previous studies have found that occupational stress can make nurses feel burnout (Liu and Aungsuroch, 2019) and can also increase the incidence of anxiety, depression, and other psychological problems, which can eventually affect their physical and mental health and quality of life (Buselli et al., 2020). However, few studies have focused on nurses in infectious disease departments specifically. We speculate that there may be a correlation between occupational stress and the quality of life of nurses in infectious disease departments in China. Therefore, our first hypothesis is:

Hypothesis 1. Occupational stress of nurses in infectious disease departments in China is negatively correlated with their quality of life.

### Psychological Resilience as a Mediator for Occupational Stress

With the rise of positive psychology, psychological resilience has become a hot research topic in both psychology and nursing. Psychological resilience is also known as mental toughness and refers to the ability to bounce back in the face of adversity, trauma, tragedy, threats, and other stresses (Foster et al., 2019). Due to the characteristics of high workloads and low social identity (Abbas et al., 2020), nurses can face pressure from their industry and society alike. In the face of this pressure, some nurses choose to resign, and some nurses to respond actively. We posit that psychological resilience is one of the determining factors in these nurses’ responses.

High levels of psychological resilience can help individuals better cope with adverse events in life so that they can maintain positive attitudes and better cope with stressful events (Foster et al., 2019; Atay et al., 2021). However, psychological resilience may be affected by the accumulation of external pressures, and In addition to psychological resilience mediating between stress and life satisfaction (Shi et al., 2015), studies have found that it can mediate between occupational stress, anxiety, and depressive symptoms as well (Jung and Baek, 2020; Serrão et al., 2021). This study therefore explores the mediating effect of psychological resilience on the relationship between occupational stress and quality of life. Hence, our second hypothesis is:

Hypothesis 2. The psychological resilience of nurses in infectious disease departments in China plays a mediating role in the relationship between occupational stress and quality of life.

## Materials and Methods

### Participants and Data Collection

Our test subjects came from 15 infectious-disease-specialized hospitals or infectious disease departments of general hospitals in China. We used the following formula to calculate the minimum sample size: *N* = (Z_α /2_)^2^*P*(1-*P*)/δ^2^, where, according to the results of our trial test, the rate of occupational stress among nurses in the infectious disease departments was *P* = 0.7; the tolerable error, δ, is 0.03, the allowable probability of a Type-I error is α = 0.05; and the critical value for our Z-statistics is Z_α /2_ = 1.96, so *N* = 896. However, due to an expectation that 10% of responses will be invalid, our minimum sample size must actually be 986.

With the help of hospital managers, 1,578 nurses in infectious disease departments who met our inclusion criteria were surveyed from January to July in 2021 through convenient sampling. Our inclusion criteria were: nurses who had worked in an infectious disease department for more than 1 year and agreed to participate in the study. Exclusion criteria were nurses-in-training and those absent on leave. Before the survey, we told the subjects the purpose and significance of the study and also assured them of anonymity and confidentiality, and our investigation took place only after we received written informed consent from the nurses. The time limit for filling out the questionnaire was 20 to 30 min, and after the nurses completed the questionnaires, research proctors withdrew them immediately. To ensure the validity of the questionnaire, the proctors confirmed in advance the completion and validity of the questionnaire with the nurses who submitted them. In addition, during the whole process of filling out the questionnaire, the research proctors maintained a certain distance from the nurses in order to avoid any possible influence from their physical presence.

### Measures

#### Occupational Stress

We measured occupational stress using the 23 items of the Effort-Reward Imbalance Questionnaire (ERI), which was developed by Professor Siegrist in 1986 (Siegrist, 1996); the Chinese version of ERI was translated by Dr. Jian Li. The ERI is a well known scale for explaining occupational stress and is extensively used among Chinese healthcare research, and it also has a high level of criterion validity (Huang et al., 2019; Kong et al., 2020; Ge et al., 2021). The ERI includes three dimensions of occupational stress: effort (E), reward (R) and overcommitment (O). Questions 1–17 are graded on a 5-point scale, for example, “I am always under time pressure because my workload is heavy”, where the answers must range from 1 (“never”) to 5 (“always”). Questions 18–23, such as “I easily get irritated by time pressure at work”, are answered on a scale of 1 (“strongly agree”) to 4 (“strongly disagree”). The formula for an ERI score is ERI = E/(R*6/11), and occupational stress is considered to be present if ERI is greater than 1. In this study, the Cronbach’s α of this scale is 0.924, and the Cronbach’s α of the three dimensions are 0.883, 0.904, and 0.711 for E, R, and O, respectively.

#### Psychological Resilience

We assessed psychological resilience using the 25-item Chinese version of the Connor-Davidson Resilience Scale (CD-RISC), which was translated into Chinese and revised by Yu and Zhang (2007). The original Connor-Davidson Resilience Scale was compiled by Connor and Davidson (2003) and consists of three dimensions of psychological resilience: strength, tenacity, and optimism. Example items are “I can adapt when there is change” and “I will do my best no matter what the result”, which are answered on a 5-point scale from 0 (“never”) to 4 (“almost always”). In this study, the Cronbach’s α of the scale was 0.932 and ranged from 0.701 to 0.905.

#### Quality of Life

We measured quality of life using the 26-item version of the World Health Organization Quality of Life Brief Scale (WHOQOL-BREF), which was translated into Chinese and revised by Hao Yuan-Tao (Fang et al., 1999). Questions 1 and 2 are independent items used to measure an individual’s overall subjective feeling of quality of life and one’s own health status, and the remaining 24 items are divided into four dimensions: physical, psychological, social relationships, and environment. Some example questions are “Do you find life interesting?” and “Do you have enough energy for daily life?” Participants respond to these questions using a 5-point scale from 1 (“strongly agree”) to 5 (“strongly disagree”). The total score for each dimension is the average score multiplied by 4, and the higher the score, the better the quality of life. The Cronbach’s α of this scale was 0.926, and the range of Cronbach’s α of the four dimensions was 0.715–0.847.

### Statistical Analysis

We first conduct descriptive statistical analysis of each variable and analyze the correlation between occupational stress, psychological resilience, and quality of life using the Pearson correlation coefficient, and we perform the comparison of quality of life among different socio-demographic subgroups using independent *t*-tests or ANOVA as appropriate. Next, we employ a two-step structural equation model (*SEM*) in order to analyze the mediating effect of psychological resilience between occupational stress and quality of life (Anderson and Gerbing, 1988; Ke-Hai et al., 2007). Specifically, we divide the measurement model and the structural model into two steps to test our hypothesis. We also run 2,000 bootstrap resamples using 95% confidence intervals (CI) to test indirect and direct effects (Cheung, 2009).

To evaluate the adequacy of our model fitting efforts, we use the chi-square test (χ^2^), the comparative fit index (CFI), the Tucker-Lewis index (TLI), the root mean square error of approximation (RMSER), and the standardized root mean square residual (SRMR). When the χ^2^ test is not statistically significant, when the CFI and TLI are both greater than 0.90, when the RMSEA is less than 0.06, and when the SRMR is less than 0.08, the model fitting effect is said to be better (Hu and Bentler, 1999; Bandalos, 2002). However, the fitting index is sensitive to the sample size; if the sample size is large enough, the data can easily reject the hypothetical model (Garnier-Villarreal and Jorgensen, 2020).

## Results

### Socio-Demographic Characteristics and Comparison of Quality of Life

With the help of hospital administrators, 1,536 out of 1,578 respondents completed our survey. Among them, 15 nurses dropped out of the survey, 27 gave incomplete surveys, resulting in an effective response rate of 97.34%. The average age of the final sample was 32.56 years (*SD* = 6.57), and the average working years were 10.58 (*SD* = 7.22). The socio-demographic characteristics of the nurses are presented in Table 1.

**TABLE 1:** Social-demographic characteristics of the participants and the comparison of quality of life (*N* = 1,536).

Variable	Category	n (%)	Mean	*SD*	*t*	*F*	*p*
**Gender**					4.208		0.040*
	Male	50 (3.3%)	51.22	6.15			
	Female	1486 (96.7%)	51.82	8.30			
**Educational level**						0.204	0.815
	Diploma	343 (22.3%)	51.73	8.71			
	Bachelor	1189 (77.4%)	51.82	8.11			
	Master	4 (0.3%)	49.27	3.95			
**Professional title**						1.435	0.239
	Junior	270 (17.6%)	51.14	7.99			
	Middle	717 (46.7%)	51.76	8.27			
	Senior	549 (35.7%)	52.17	8.30			
**Employment type**						0.101	0.904
	Contract	1178 (76.7%)	51.75	8.08			
	Permanent	273 (17.8%)	51.99	8.97			
	Other	85 (5.5%)	51.86	8.01			
**Marital status**						2.618	0.050
	Unmarried	396 (25.8%)	50.85	8.65			
	Married	1112 (72.4%)	52.10	8.00			
	Divorced	24 (1.5%)	52.90	11.25			
	Widowed	4 (0.3%)	55.02	2.94			
**Children status**					2.909		0.088
	Have a child	1007 (65.6%)	52.22	8.05			
	Not have a child	529 (34.4%)	51.00	8.53			
**Monthly income**						7.210	0.000**
**(RMB)**	≤3000	133 (8.7%)	50.09	8.05			
	3001–5000	552 (35.9%)	51.34	8.26			
	5001–8000	629 (40.9%)	51.83	7.85			
	≥8001	222 (14.5%)	53.86	8.99			

*p < 0.05; **p < 0.01.

We performed the comparison of quality of life among different sociol-demographic subgroups using independent *t*-tests or ANOVA as appropriate (Table 1). For quality of life, we observed statistically significant differences in gender (*p* = 0.040) and monthly income (*p* = 0.000) but found no significant differences in the quality of life scores among nurses with different education levels, professional titles, employment types or marital status.

### Descriptive Analysis of Occupational Stress, Psychological Resilience, and Quality of Life Scores

Among the 1,536 nurses in infectious disease departments in China, 1,355 nurses experienced occupational stress (ERI ratio > 1), with a detection rate of 88.2%. Table 2 shows the scores of the nurses in occupational stress, psychological resilience, and quality of life across all dimensions.

**TABLE 2:** Descriptive statistics of all measures used in the current study.

		Mean	*SD*	Min.	Max.
1	Effort	15.63	5.30	6.00	30.00
2	Reward	20.06	8.63	11.00	55.00
3	Overcommitment	16.10	2.60	6.00	24.00
4	Occupational stress	51.79	14.36	23.00	106.00
5	ERI ratio	1.54	0.50	0.43	4.00
6	Strength	19.75	4.86	0.00	32.00
7	Tenacity	28.36	8.16	0.00	52.00
8	Optimism	7.95	2.59	0.00	16.00
9	Psychological resilience	56.06	14.19	0.00	100.00
10	Physical	12.57	1.95	4.00	19.43
11	Psychological	13.51	2.64	4.00	20.00
12	Social relationship	13.57	2.58	4.00	20.00
13	Environment	12.15	2.38	4.00	20.00
14	Quality of life	51.80	8.23	16.00	79.43

*The possible score range for all measures is 0–100. N = 1536.*

### Correlation Analysis

The correlation analysis results among the variables we studied are shown in Table 3. The results show that occupational stress is negatively correlated with psychological resilience (*r* = −0.28, *p* < 0.01) and quality of life (*r* = −0.44, *p* < 0.01). In addition, psychological resilience is positively correlated with quality of life (*r* = 0.55, *p* < 0.01), and correlation analysis results show that each dimension is significantly correlated with other dimensions of the same structure, which indicates that the measurement we used has good convergent validity.

**TABLE 3:** Correlations among study variables.

		1	2	3	4	5	6	7	8	9	10	11	12
1	Effort												
2	Reward	0.672**											
3	Overcommitment	0.551**	0.451**										
4	Occupational stress	0.873**	0.931**	0.656**									
5	Strength	−0.260**	−0.280**	−0.074**	−0.278**								
6	Tenacity	−0.247**	−0.238**	−0.085**	−0.250**	0.784**							
7	Optimism	−0.200**	−0.217**	−0.065*	−0.216**	0.660**	0.605**						
8	Psychological resilience	−0.268**	−0.273**	−0.086**	−0.278**	0.914**	0.954**	0.756**					
9	Physical	−0.388**	−0.333**	−0.265**	−0.391**	0.483**	0.499**	0.376**	0.521**				
10	Psychological	−0.374**	−0.354**	−0.264**	−0.398**	0.485**	0.496**	0.420**	0.528**	0.719**			
11	Social relationship	−0.258**	−0.271**	−0.169**	−0.288**	0.329**	0.348**	0.290**	0.366**	0.579**	0.632**		
12	Environment	−0.402**	−0.409**	−0.282**	−0.445**	0.438**	0.442**	0.409**	0.479**	0.669**	0.736**	0.602**	
13	Quality of life	−0.409**	−0.395**	−0.282**	−0.439**	0.500**	0.514**	0.433**	0.546**	0.842**	0.902**	0.827**	0.872**

N = 1536. *p < 0.05, **p < 0.01.

### Testing the Research Hypotheses

To test our hypotheses, we first use the confirmatory factor method to evaluate the fitting effect of our measurement model, which includes three dimensions: occupational stress, psychological resilience, and quality of life. The results show that the three-factor model has a good fitting effect according to the criteria discussed above. Specifically, χ^2^ = 41.052, *df* = 20, χ^2^/*df* = 2.053 < 3, CFI = 0.997 > 0.900, TLI = 0.994 > 0.900, RMSEA = 0.021 < 0.06, 90% CI: 0.015–0.038, and SRMR = 0.012 < 0.08.

**FIGURE 1:**
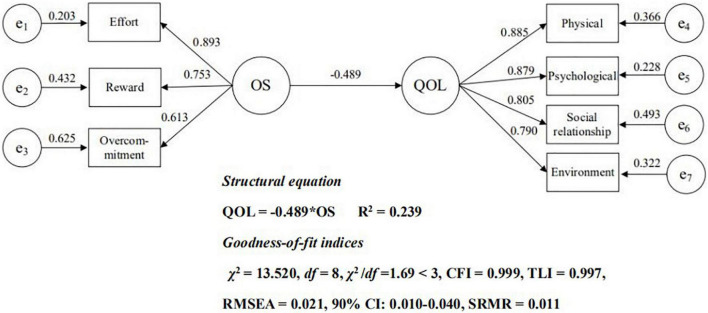
Direct Effect Model. OS, occupational stress; e_1_–e_3_, manifest variables of the three dimensions of occupational stress; QOL, quality of life; e_4_–e_7_, manifest variables of the three dimensions of quality of life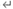.

Next, we test a direct effect model to determine whether the occupational stress of the nurses directly affects their quality of life (Hypothesis 1). The results show that the direct effect model fits well, with χ^2^ = 13.520, *df* = 8, χ^2^/*df* = 1.69 < 3, CFI = 0.999 > 0.90, TLI = 0.997 > 0.900, RMSEA = 0.021 < 0.060, 90% CI: 0.000–0.040, and SRMR = 0.011 < 0.080, and all parameters were statistically different (*p* < 0.001). We present the path coefficients that directly affect the model in Figure 1. Here we see that occupational stress is negatively correlated with quality of life (β = −0.489, *p* < 0.001).

Next, we conducted 2,000 bootstrap samples to construct a 95% confidence interval for the overall direct effect of occupational stress on quality of life. These results show that the 95% confidence interval of the total direct effect is (−0.207, −0.156), and we find that occupational stress explains 24% of the difference in quality of life between nurses.

**FIGURE 2:**
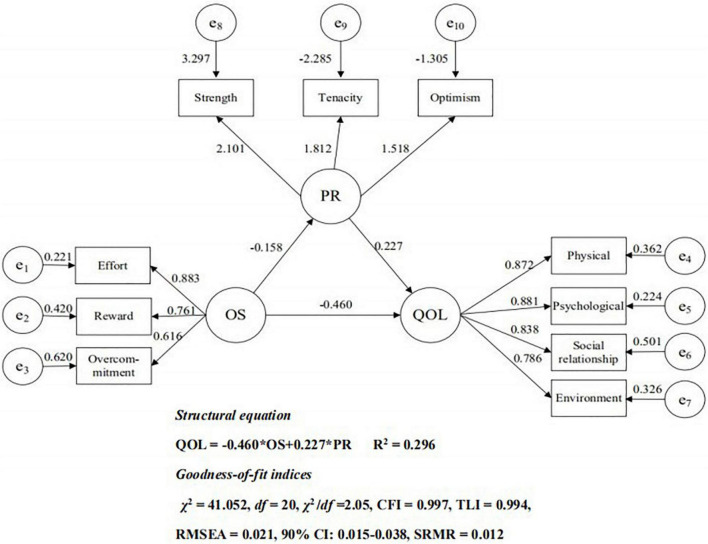
Mediation Model. OS, occupational stress; e_1_–e_3_, manifest variables of the three dimensions of occupational stress: QOL, quality of life: e_4_–e_7_, manifest variables of the three dimensions of quality of life; PR, psychological resilience; e_8_–e_10_, manifest variables of the three dimensions of psychological resilience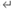.

Finally, we test the mediating model to determine whether psychological resilience mediates the relationship between occupational stress and quality of life (Hypothesis 2). The results once again show that the direct effect model has a good fit, with χ^2^ = 41.052, *df* = 20, χ^2^/*df* = 2.05 < 3, CFI = 0.997 > 0.90, TLI = 0.994 > 0.90, RMSEA = 0.021 < 0.06, 90% CI: 0.015–0.038, and SRMR = 0.012 < 0.08, and each parameter is statistically significant (*p* < 0.001). Figure 2 depicts the mediation effect model. We find a negative correlation between occupational stress and psychological resilience (β = −0.158, *p* < 0.001) and that psychological resilience has a positive effect on quality of life (β = 0.227, *p* < 0.001).

Furthermore, occupational stress is still significantly associated with quality of life (β = −0.460, *p* < 0.001) in this model. That is, we find that psychological resilience plays a partial mediating role. In order to construct the 95% confidence interval of the indirect effect of occupational stress on quality of life through psychological resilience, we again conduct 2,000 bootstrap samples, and the bootstrap results show that the 95% confidence interval of the mediating effect is (0.027, 0.426). Here, occupational stress and psychological resilience together explain 30% of the difference in quality of life between nurses, and we find that the indirect effect of occupational stress on quality of life accounted for 7.3% of the total effect (total effect = −0.496, indirect effect = −0.036).

## Discussion

### The Status of Occupational Stress, Psychological Resilience, and Quality of Life

According to the ERI scores of the nurses, 88.2% stated that they experienced an effort–reward imbalance, which is higher than in Deng et al. (2021) study of 370 community health workers (78.39%). This percentage is also higher than that obtained from the study of 1,107 nurses by Kong et al. (2020) (26.50%). One explanation for these results may be that nurses in infectious disease departments in China work in a more specialized environment and face more patients, which may result in greater pressure from work.

In this study, the “effort” dimension of occupational stress refers specifically to the fact that due to the nature of infectious disease departments, the disinfection and isolation requirements of the working environment are strict. In addition, infectious diseases can have characteristics such as acute onset, serious symptoms, strong infectivity, and high risk of occupational exposure, which require higher qualifications for working ability and psychological quality from nurses (Brooks et al., 2018).

The “reward” dimension includes money, respect, and promotion opportunities. Our results show that nurses in infectious disease departments in China scored higher on the “reward” dimension, and this indicates that these nurses feel that they are not being respected and rewarded enough at work. When nurses perceive their jobs to be high-effort and low-reward, they can develop a sense of effort and reward imbalance. When this sense of imbalance reaches a certain threshold, it can show up in the nurses’ answers to our questionnaires, and we may judge that they experience occupational stress. One recent source of occupational stress may be the fact that nurses in infectious disease departments in China have taken on many additional front-line tasks during the COVID-19 pandemic. This extra workload may make them more prone to occupational stress due to fatigue and even fear (Mehta et al., 2021).

Our study shows that the total score of psychological resilience for nurses in infectious disease departments in China was 56.06 (*SD* = 14.19), which is considered to be at a moderate level. However, compared with the research results of Guo et al. (2018) on 1,061 nurses from grade III hospitals in Hunan Province, the total scores of psychological resilience from our study (*t* = −21.292, *p* < 0.001), strength (*t* = −17.236, *p* < 0.001), tenacity (*t* = −18.052, *p* < 0.001), and optimism (*t* = −27.432, *p* < 0.001) were significantly lower, and this relatively lower psychological resilience of nurses in infectious disease departments in China may be due to any of the following reasons. First, it may be related to the special work nature of infectious disease departments, where occupational exposure risks are high. All patients with whom nurses in these departments are in contact every day are contagious to some extent. In addition, special protective appliances are often used in nursing operations, and their use can increase the difficulty of the operations and also cause certain psychological pressure to nurses (Yuan et al., 2021). Second, high workload can lead to fatigue, frequent night shifts can cause shift work disorder, and short-term poor amounts of rest and recovery can affect mental health (Labrague, 2021).

For quality of life, our results show that the average scores of female nurses are higher than those of male nurses. In China, the now “two-child policy” has increased the maternity leave of female nurses compared to the “one-child policy”, and caused male nurses to correspondingly undertake more work. However, although male nurses make up only a modest percentage of the nursing workforce, they are more likely to be valued by hospital leaders. Additionally, they will on average be more severe with themselves in order to do better, thereby increasing the pressure from work. Male nurses also receive literally heavier workloads in clinical work due to their superior strength compared to females. Men also typically have larger family duties and face greater pressure at home, which can easily impair their feeling of well-being, resulting in a worse quality of life than female nurses.

For quality of life in terms of monetary compensation, however, we find that the higher the monthly pay, the better the quality of life for nurses. The larger the income, the greater the sense of personal worth as well. When they receive more money for their time, people have more options for meeting their basic needs, pursuing a richer life experience, and improving their quality of life.

The scores of the quality of life of nurses in infectious disease departments in our study in the fields of physiology (*t* = −50.93; *p* < 0.001), psychology (*t* = −5.65; *p* < 0.001), and social relationships (*t* = −5.51; *p* < 0.001) were all significantly lower than the Chinese norm (Fang et al., 1999). However, there was no significant difference between the scores for the environmental domain. Sometimes, the symptoms of infectious diseases are not easy to detect, and family members often lack relevant knowledge of nursing, isolation, and protection. Hence nurses must invest relatively more energy in keeping their families safe from their own workplace hazards, decreasing their quality of life. Additionally, there is such a large shortage of nursing staff in China (Twigg and McCullough, 2014), that nurses are always overloaded with work.

Furthermore, we find that the score in the field of environment was the lowest, which is consistent with Kowitlawkul et al. (2019) research. This result may be because infectious disease departments have high requirements for the environment and limit the number of companions that can enter, which may lead nurses to face negative emotions of family members. In addition, busy work schedules and high pressure reduce nurses’ participation in leisure and entertainment activities after work, which can also affect their quality of life.

### The Direct Influence of Occupational Stress on Quality of Life

Our results provide evidence that the occupational stress on nurses in infectious disease departments in China has a negative impact on their quality of life, consistent with our Hypothesis 1 and also with the findings of other scholars (Chegini et al., 2019; Ruiz-Fernández et al., 2020b). In addition, studies have shown that the lower the occupational stress, the better the quality of life. Nurses in infectious disease departments work intensely, and when they’re in a state of tension, they can experience a decline in physical health. Studies have found that occupational stress factors can affect the cardiovascular and immune systems, as well as nurses’ physiology and psychology (Buselli et al., 2020).

In addition, the nature of infectious disease departments requires nurses to have strong adaptability and tolerance to emergencies. When nurses feel that they’re giving more but not getting more in return, they develop a sense of imbalance, a higher sense of occupational stress. In this way, nurses can become less interested in work and may not feel a sense of accomplishment from their jobs What’s more, the public has a fear of infectious diseases (Anjum et al., 2020) that can lead to prejudice against nurses in infectious disease departments. This prejudice can bring psychological pressure to nurses and thus affect their social relations and social support. Ultimately, this can reduce nurses’ happiness and affect their quality of life.

### The Mediating Effect of Psychological Resilience on Occupational Stress and Quality of Life

The results of our SEM show that psychological resilience partially mediates the relationship between occupational stress and quality of life among nurses in infectious disease departments in China. The indirect effect of occupational stress on quality of life through psychological resilience accounts for 7.3% of the total effect, indicating that our constructed hypothesis model had an explanatory power for the quality of life of the nurses. Occupational stress not only directly and negatively predicts quality of life but also indirectly and negatively predicts quality of life with psychological resilience as a mediating variable. Our study finds that nurses’ psychological resilience can positively predict quality of life, which is consistent with existing relevant research results (Atay et al., 2021; Keener et al., 2021).

Their high degree of occupational stress means that nurses feel greater work pressure and a greater imbalance between work effort and reward. Some studies have found that ERI is positively correlated with fatigue in occupational groups, including nurses (Sembajwe et al., 2012; Huang et al., 2019), and a strong sense of imbalance can affect the quality of the nurse’s work and produce negative emotions that can result in a reduction in mental tenacity, strength, and optimism (Liu et al., 2020). Occupational stress can also lead to physical discomfort by altering a nurse’s psychological state (Hong and Lee, 2016). A low level of psychological resilience in nurses can prevent them from actively taking measures to relieve pressure as well (Delgado et al., 2017; Walpita and Arambepola, 2020), and this can affect their normal communication with colleagues and friends. Nurses are more likely to have anxiety, depression, and other negative psychological conditions that can reduce their job satisfaction and overall happiness and ultimately lead to a decline in their quality of life.

Studies have found that psychological resilience can be used as a protective factor for individuals to cope with occupational stress (Babić et al., 2020) and can also help individuals effectively cope with pressure and relieve negative emotions (Yörük and Güler, 2021). Furthermore, by using psychological resilience as a self-protection mechanism, nursing staff can maintain their mental health through self-psychological adjustment, cope with pressure at work, and relieve occupational stress, helping to maintain a better quality of life (Keener et al., 2021).

## Conclusion

Our study focused on occupational stress, psychological resilience, and the quality of life of nurses in infectious disease departments in China. Through statistical analysis of our survey data, we find that the occupational stress level of nurses in infectious disease departments is high and that their overall quality of life is low. In addition we find that occupational stress can directly and negatively predict quality of life but that the relationship between occupational stress and quality of life is partially mediated by psychological resilience, which itself had a positive predictive effect on quality of life.

### Implication for Nursing Management

Nursing managers can benefit by paying attention to not only the direct effect of occupational stress on quality of life but also to the mediating effect of psychological resilience. First, active and extensive publicity and education may cause the public to have a more sympathetic understanding of infectious diseases and thereby reduce people’s prejudice against nurses. Second, management can improve the security and reward system, make more reasonable and flexible scheduling and make the division of tasks clear and reasonable. Head nurses can try to rationalize work demands (workload, overtime, etc.) and work rewards (fairness, respect, support, etc.) to achieve a balance between effort and reward. Third, managers can maintain communication with nurses and provide channels to express negative emotions. In addition, managers can conduct regular mindfulness therapy training (Lee et al., 2019), resilience training (Babanataj et al., 2019), psychological lectures, and other methods to help nurses relieve pressure and to improve their psychological resilience.

### Limitations

There are certain limitations to our study. First, we employed the convenience sampling method in this study, which may bring with it selection bias. Second, we explore the relationship between relevant variables through static, cross-sectional studies only. Future studies can carry out practical interventions on relevant variables and evaluate their effects.

## Data Availability Statement

The raw data supporting the conclusions of this article will be made available by the authors, without undue reservation.

## Ethics Statement

The studies involving human participants were reviewed and approved by the Ethics Committee of Xijing Hospital of Fourth Military Medical University. The patients/participants provided their written informed consent to participate in this study.

## Author Contributions

JY and CW contributed equally to the research design, data analysis, and writing of the manuscript. YD and SH contributed to the distribution and collection of the questionnaires. LS contributed to the design of the research and provided guidance for statistical analysis statistics. HL ensured that the descriptions were accurate and agreed upon by all authors. All authors reviewed the manuscript.

## Conflict of Interest

The authors declare that the research was conducted in the absence of any commercial or financial relationships that could be construed as a potential conflict of interest.

## Publisher’s Note

All claims expressed in this article are solely those of the authors and do not necessarily represent those of their affiliated organizations, or those of the publisher, the editors and the reviewers. Any product that may be evaluated in this article, or claim that may be made by its manufacturer, is not guaranteed or endorsed by the publisher.
